# Clinical independent prognostic factors and overall survival prognostic nomogram for intracranial subependymoma: A SEER population-based analysis 2004–2016

**DOI:** 10.3389/fonc.2022.939816

**Published:** 2022-08-22

**Authors:** Zibin Zhang, Xiaojun Pang, Yuyu Wei, Qingping Lv, Xuhong Jin, Huai Chen

**Affiliations:** Department of Neurosurgery, Affiliated Hangzhou Chest Hospital, Zhejiang University School of Medicine, Hangzhou, China

**Keywords:** intracranial subependymoma, SEER, nomogram, surgery, prognosis

## Abstract

**Purpose:**

This study was launched to ascertain the independent prognostic factors influencing the overall survival (OS) prognosis of intracranial subependymoma and construct a prognostic model to predict OS time.

**Materials and methods:**

We collected data from patients with intracranial subependymoma, including treatment data, follow-up data, and clinical and pathological characteristics from the SEER database within 2004 to 2016, and patients were randomly classified into training and validation cohorts. Univariate and multivariate analyses were applied to the training group through building a Cox proportional hazards model. According to the results of multivariate analysis, we established a nomogram to forecast the OS rate of the per-case patient graphically, then calculated the accuracy of verification in both training and validation cohorts by concordance index (C-index). Univariate and multivariate analyses were used for different subgroups of unoperated versus operated, gross total resection (GTR), subtotal resection (STR), and biopsy after using the propensity score matching (PSM) analyses.

**Results:**

A total of 667 patients were enrolled, and we randomly assigned 535 patients (80.21%) into the training cohort and 132 patients (19.79%) into the validation cohort. Age [hazard ratio (HR) = 6.355; 95% confidence interval (CI), 2.240–18.029; *p* = 0.001] and sex (HR = 0.475; 95% CI, 0.232–0.974; *p* = 0.042) were the independent prognostic factors in the training cohort. On the basis of age and sex, the nomogram was established to predict the OS for every patient (C-index = 0.733 ± 0.065 in the training cohort and 0.850 ± 0.065 in the validation cohort), and calibration plots reflected the reliability of the nomogram. Age, gender, or laterality was the independent prognostic factor for OS in the different matched subgroups of unoperated versus operated, GTR, STR, and biopsy. Surgical treatment, race, year of diagnosis, insurance, tumor location, tumor size, pathology, tumor grade, and radiation were not statistically significantly different in OS for subependymoma in our research.

**Conclusion:**

Age and sex were the independent prognostic variables for OS in intracranial subependymoma. According to our research, we should not be more inclined to choose conservative or surgical treatment. Nonetheless, the information that we present might be useful to suggest potential hypotheses to be tested in the clinical research setting.

## Introduction

Subependymoma is a neoplasm with a low incidence and low degree of malignancy (1–3).

Middle-aged and elderly men were the most affected age group by this type of cancer (4). The location of neoplasms was more likely to occur in the ventricle system than in the brain parenchyma or spinal cord (5). Generally speaking, surgical intervention has been recommended once symptoms occur, such as hydrocephalus (6), and conservative treatment has been used for incidental asymptomatic subependymoma. However, there is no detailed analysis of the different prognosis between conservative and surgical treatment including biopsy, STR, and GTR. Consequently, our study was launched to ascertain the independent factors influencing the OS prognosis of intracranial subependymoma and construct a prognostic model to predict OS time through exploring the SEER database.

## Methods

### Data

The data of 667 patients with intracranial subependymoma were investigated, including treatment and follow-up data, and clinical and pathological characteristics between 2004 and 2016 from the SEER (1975–2016 varying) database, by the SEER*Stat software (version 8.3.9.2).

This study’s inclusion criteria included the following: (1) patient’s ICD-O-3 histology codes in accordance with 9383/0 (subependymoma, benign), 9383/1 (subependymoma), or 9383/2(subependymoma, malignant); and (2) patients with definite information on the vital status and OS.

**Figure 1:**
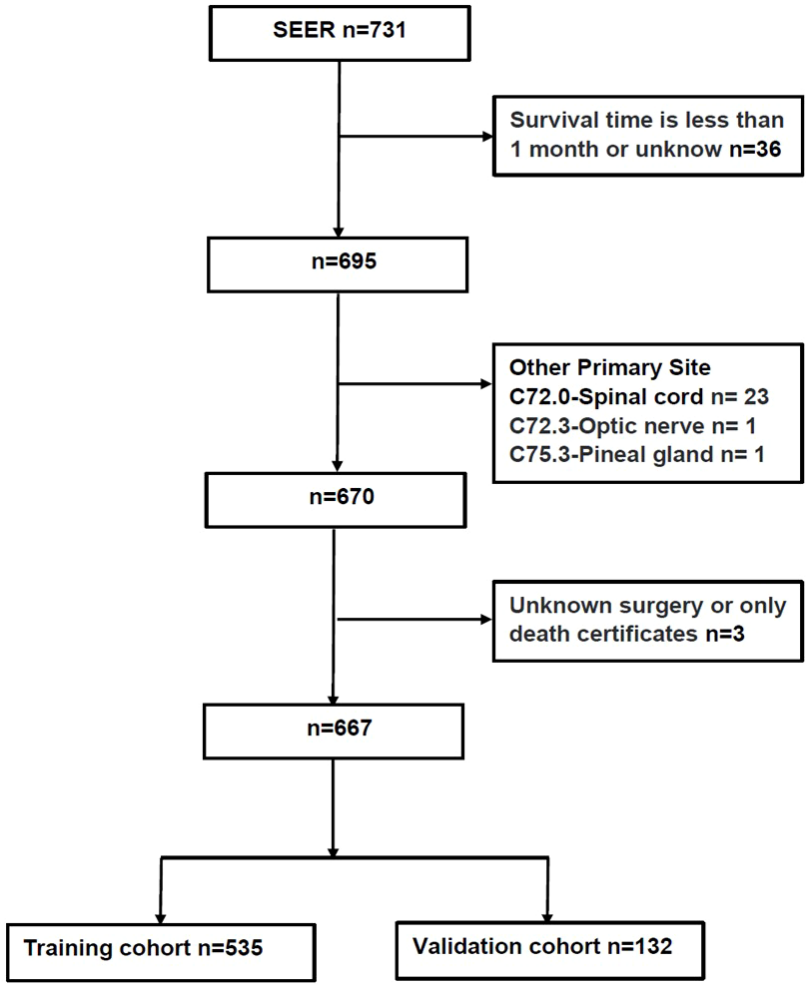
Method of retrieving data from the data base.

This study’s exclusion criteria included the following: (1) patients with no specific survival time or with an OS time of less than 1 month; (2) tumor location involving pineal gland (C75.3), spinal cord (C72.0), or optic nerve (C72.3); and (3) the patient had no other specific information or unknown treatment, only a death certificate or an autopsy. The data on age, sex, race, year of diagnosis, insurance, marital status, primary site, tumor size, pathology, grade, laterality, primary site surgery, radiation, vital status, and OS were obtained. The method of retrieving data from the database is shown in **Figure 1**.

### Endpoints

We used OS defined from the time of diagnosis to death or last investigation as the primary endpoint.

### Statistical analysis

The whole sample was divided into a training and a validation cohort. Age, a continuous variable, was changed to an ordered classification variable. Disordered classification variables were analyzed by using the *χ* ^2^ test or Fisher’s exact test, including sex, race, year of diagnosis, marital status, primary site, tumor size, grade, laterality, primary site surgery, and radiation. Ordered classification variables were analyzed by using the Mann-Whitney *U* test, including age, insurance, and pathology. Different survival rates of variables were graphically evaluated by using the Kaplan-Meier method.

The Cox proportional hazards model was used to perform univariate and multivariate analyses on the training group. According to clinical independent prognostic factors, a nomogram predicting survival probabilities at 3, 5, and 10 years for subependymoma patients was constructed through using the rms package in R (version 4.1.2) in the training group. The model’s C-index, and 3 - and 5-year calibration curves in the training cohort were calculated. The nomogram was further validated by calculating C-index in the validation cohort.

The Cox proportional hazards model method and PSM were used in different subgroups of 667 patients including unoperated versus operated, GTR, STR, and biopsy. A logistic regression model was constructed with operation status as the dependent variable for calculating the propensity scores. One-to-one matching without replacement was performed using the nearest-neighbor match on the logit of the propensity score for confounding factors (derived from age, sex, race, marital status, primary site, tumor size, pathology, grade, laterality, and radiation). The χ^2^ test, Fisher’s exact test, and Mann-Whitney *U* test were used to inspect the statistical differences of subgroups before and after matching. Cox proportional hazards model was used to perform univariate and multivariate analyses of various subgroups’ data after PSM. Equilibrium of covariables between subgroups was indicated by *p* **>** 0.05.

Various statistical methods were finished in this paper by SPSS (SPSS 26.0, IBM, Inc., Armonk, NY, United States) and R software (R 4.1.2, Vienna, Austria). The *p*-value < 0.10 of the factor in the univariate analysis was included in the multivariate analysis. Two-tailed *p*-value < 0.05 was indicated statistically significant (7).

## Results

### Patient characteristics

Our study included 667 cases of intracranial subependymoma, which randomly assigned 535 patients (80.21%) into the training cohort and 132 patients (19.79%) into the validation cohort (**Figure 1**). The median OS for all of patients, training cohort, and validation cohort was 56 months [interquartile range (IQR), 24–93], 56 months (IQR, 22–93), and 57 months (IQR,25–90), respectively (**Table 1**).

**Table 1:** Details of patients with subependymoma.

Characteristics	Total *n* = 667	Training cohort *N* = 535	Validation cohort *N* = 132
Primary site surgery			
No surgery	243 (36.43%)	196 (36.64%)	47 (35.61%)
Surgery NOS or excisional biopsy	101 (15.14%)	86 (16.07%)	15 (11.36%)
STR	96 (14.39%)	77 (14.39%)	19 (14.39%)
GTR	227 (34.03%)	176 (32.90%)	51 (38.64%)
**Age (years)**			
0–39	126 (18.89%)	99 (18.50%)	27 (20.45%)
40–59	331 (49.63%)	266 (49.72%)	65 (49.24%)
≥60	210 (31.48%)	170 (31.78%)	40 (30.30%)
**Sex**			
Male	472 (70.76%)	375 (70.09%)	97 (73.48%)
Female	195 (29.23%)	160 (29.91%)	35 (26.52%)
**Race**			
White	584 (87.56%)	470 (87.85%)	114 (86.36%)
Black	37 (5.55%)	28 (5.23%)	9 (6.82%)
Others/Unknown	46 (6.90%)	37 (6.92%)	9 (6.82%)
**Year of diagnosis**			
4–9	230 (34.48%)	185 (34.58%)	45 (34.09%)
10–16	437 (65.52%)	350 (65.42%)	87 (65.91%)
**Insurance**			
Uninsured/unknown/blank	131 (19.64%)	102 (19.07%)	29 (21.97%)
Insured/no specifics	467 (70.01%)	376 (70.28%)	91 (68.94%)
Any Medicaid	69 (10.34%)	57 (10.65%)	12 (9.09%)
**Marital status**			
Married (including common law)	394 (59.07%)	185 (34.58%)	82 (62.12%)
Other	273 (40.93%)	350 (65.42%)	50 (37.88%)
**Primary site**			
Ventricle, NOS	347 (52.02%)	282 (52.71%)	65 (49.24%)
Brain stem	199 (29.84%)	153 (28.60%)	46 (34.85%)
Other	121 (18.14%)	100 (18.69%)	21 (15.91%)
**Tumor size (cm)**			
<2	216 (32.38%)	174 (32.52%)	42 (31.82%)
2–4	202 (30.28%)	155 (28.97%)	47 (35.61%)
≥4	62 (9.30%)	52 (9.72%)	10 (7.58%)
Unknown/blank	187 (28.04%)	154 (28.79%)	33 (25.00%)
**Pathology**			
Benign	6 (0.90%)	5 (0.93%)	1 (0.76%)
Subependymoma	656 (98.35%)	526 (98.32%)	130 (98.48%)
Malignant	5 (0.75%)	4 (0.75%)	1 (0.76%)
**Grade**			
Well differentiated	50 (7.50%)	41 (7.66%)	9 (6.82%)
Moderately differentiated	10 (1.50%)	9 (1.68%)	1 (0.76%)
Undifferentiated	1 (0.15%)	1 (0.19%)	0 (0.00%)
Unknown	606 (90.85%)	484 (90.47%)	122 (92.42%)
**Laterality**			
Left-origin of primary	77 (11.54%)	63 (11.78%)	14 (10.61%)
Right-origin of primary	86 (12.89%)	69 (12.90%)	17 (12.88%)
Not a paired site	484 (72.71%)	386 (72.15%)	98 (74.24%)
Paired or bilateral	20 (3.00%)	17 (3.18%)	3 (2.23%)
**Radiation**			
None/Unknown	640 (95.95%)	513 (95.89%)	127 (96.21%)
Yes	27 (4.05%)	22 (4.11%)	5 (3.79%)
**Vital status**			
Alive	596 (89.36%)	478 (89.35%)	118 (89.39%)
Dead	71 (10.64%)	57 (10.65%)	14 (10.61%)
**OS (M)**	56 (24-93)	56 (22-93)	57 (25-90)

GTR, gross total resection; STR, subtotal resection; OS, overall survival.

### The survival factors of the training cohort

**Figure 2:**
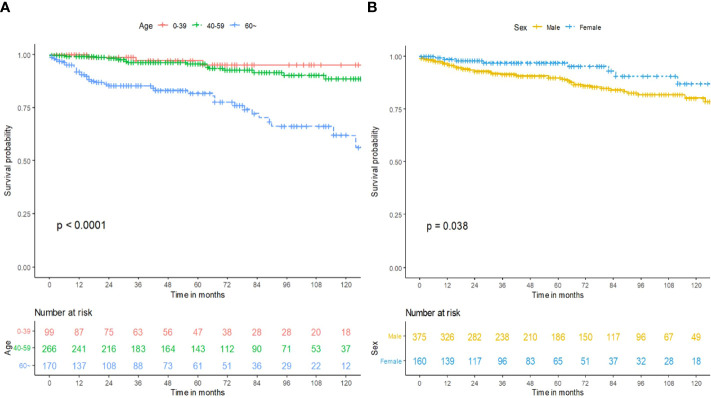
Prognosis of intracranial subependymoma for OS in the training cohort. **(A)** OS between the different age groups. **(B)** OS between the different sex groups. OS, overall survival.

The survival curves of age (*p* < 0.0001; **Figure 2A**) and sex (*p* = 0.038; **Figure 2B**) were compared using a log-rank test. The Cox proportional hazards model was used to perform univariate and multivariate analysis for the training cohort. As exhibited in **Table 2**, age (*p* < 0.001) and sex were (*p* = 0.042) independent prognostic predictors. Patients who were male or more than 60 years of age had less OS time compared with patients who were female or less than 60 years of age. Race, year of diagnosis, insurance, marital status, primary site, tumor size, pathology, grade, laterality, primary site surgery, and radiation had no statistically significant differences in OS for subependymoma in our research (**Table 2**).

**Table 2:** Training cohort characteristics.

Characteristics	Value *N* = 535	Univariate analysis			Multivariate analysis		
		HR	95% CI	*p*-value	HR	95% CI	*p*-value
Primary site surgery							
No surgery	196 (36.64%)	Reference			Reference		
Surgery NOS or excisional biopsy	86 (16.07%)	0.725	0.337–1.560	0.411	0.953	0.438–2.076	0.904
STR	77 (14.39%)	0.930	0.444–1.948	0.847	1.061	0.504–2.235	0.876
GTR	176 (32.90%)	0.566	0.292–1.096	0.091	0.799	0.405–1.574	0.516
**Age (years)**							
0–39	99 (18.50%)	Reference			Reference		
40–59	266 (49.72%)	1.570	0.528–4.672	0.417	1.484	0.496–4.442	0.481
≥60	170 (31.78%)	6.821	2.420–19.226	<0.001	6.355	2.240–18.029	0.001
**Sex**							
Male	375 (70.09%)	Reference			Reference		
Female	160 (29.91%)	0.478	0.235–0.975	0.043	0.475	0.232–0.974	0.042
**Race**							
White	470 (87.85%)	Reference					
Black	28 (5.23%)	0.986	0.308–3.159	0.981			
Others/Unknown	37 (6.92%)	0.210	0.029–1.522	0.123			
**Year of diagnosis**							
4–9	185 (34.58%)	Reference					
10–16	350 (65.42%)	1.174	0.639–2.156	0.606			
**Insurance**							
Uninsured/unknown/blank	102 (19.07%)	Reference					
Insured/no specifics	376 (70.28%)	1.484	0.755–2.914	0.252			
Any Medicaid	57 (10.65%)	1.641	0.606–4.441	0.330			
**Marital status**							
Married (including common law)	185 (34.58%)	Reference					
Other	350 (65.42%)	1.270	0.752–2.145	0.372			
**Primary site**							
Ventricle, NOS	282 (52.71%)	Reference					
Brain stem	153 (28.60%)	1.221	0.678–2.198	0.507			
Other	100 (18.69%)	1.058	0.515–2.172	0.879			
**Tumor size (cm)**							
<2	174 (32.52%)	Reference					
2–4	155 (28.97%)	0.907	0.462–1.781	0.778			
≥4	52 (9.72%)	0.921	0.342–2.481	0.870			
Unknown/blank	154 (28.79%)	1.208	0.627–2.328	0.573			
**Pathology**							
Benign	5 (0.93%)	Reference					
Subependymoma	526 (98.32%)	0.564	0.078–4.082	0.570			
Malignant	4 (0.75%)	0.801	0.050–12.861	0.876			
**Grade**							
Well differentiated	41 (7.66%)	Reference					
Moderately differentiated	9 (1.68%)	NA	NA	NA			
Undifferentiated	1 (0.19%)	NA	NA	NA			
Unknown	484 (90.47%)	0.734	0.315–1.711	0.474			
**Laterality**							
Left-origin of primary	63 (11.78%)	Reference					
Right-origin of primary	69 (12.90%)	0.622	0.216–1.797	0.381			
Not a paired site	386 (72.15%)	0.684	0.320–1.463	0.328			
Paired or bilateral	17 (3.18%)	0.492	0.062–3.936	0.504			
**Radiation**							
None/unknown	513 (95.89%)	Reference					
Yes	22 (4.11%)	1.145	0.358–3.666	0.820			
**Vital status**							
Alive	478 (89.35%)						
Dead	57 (10.65%)						
**OS (M)**	56 (22-93)						

HR, hazard ratio; CI, confidence interval; GTR, gross total resection; STR, subtotal resection; OS, overall survival; NA, not available.

The median OS time was 56 months (interquartile range, IQR 22–93). The Cox proportional hazards model was used to perform univariate and multivariate analyses on the training group.

### Construction and validation of the nomogram

**Figure 3:**
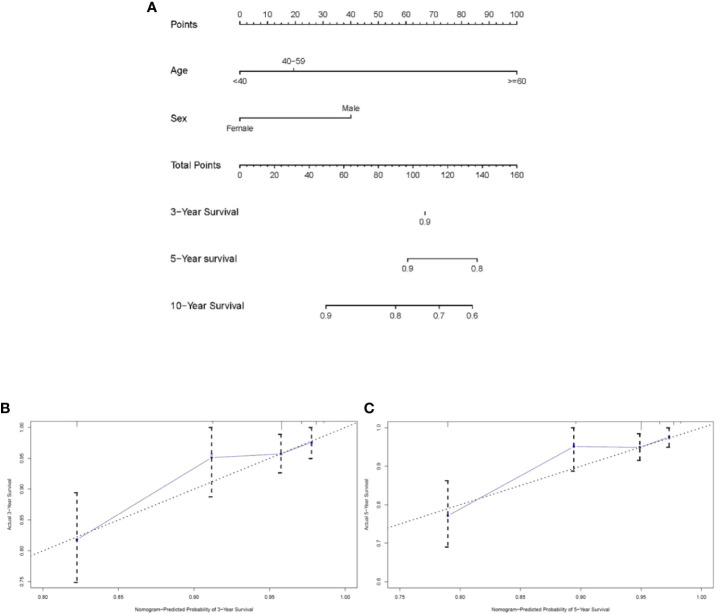
Nomogram analyses for patients with intracranial subependymoma. **(A)** A nomogram for predicting 3-, 5- and 10-year OS of patients. **(B)** Calibration curve of the nomogram predicting 3-year OS in training cohort. **(C)** Calibration curve of the nomogram predicting 5-year OS in training cohort. OS, overall survival.

The Cox proportional hazards model uncovered two significant factors that were used to build the nomogram in the training cohort at last (**Figure 3A**). As exhibited in **Figures 3B, C**, calibration diagrams complement the internal validation of training queue. The C-index of the training cohort was 0.733 ± 0.065. The C-index and calibration plots confirmed the dependability of the nomograms. Then, the C-index of the validation cohort was 0.850 ± 0.065. Therefore, the 3-year, 5-year, and 10-year predictions of OS by the nomograms were reliable.

### Different subgroups after COX regression analysis and PSM

We performed different subgroup analyses to determine whether surgery was an independent predictor for OS. After PSM of 667 patients in unoperated versus operated, 210 non-operative patients were matched with 210 surgical patients (**Tables 3**, **S1**). In the matched cohort, there was no significant difference in OS between the non-surgical and surgical groups (HR = 0.788; 95% CI, 0.457–1.359; *p* = 0.391; **Table 3**). In the multivariable regression analysis, age (HR = 8.870; 95% CI, 2.106–22.410; *p* = 0.001; **Table 3**) and sex (HR = 0.380; 95% CI, 0.170–0.846; *p* = 0.018; **Table 3**) were independent risk prognostic factors for OS.

**Table 3:** The characteristics of 420 patients from the 667 patients grouped according to no surgery and surgery after PSM.

Characteristics	Value *N* = 420	Univariate analysis			Multivariate analysis		
		HR	95% CI	*p*-value	HR	95% CI	*p*-value
Primary site surgery							
No surgery	210 (50.00%)	Reference					
Surgery	210 (50.00%)	0.788	0.457–1.359	0.391			
**Age (years)**							
0–39	77 (18.33%)	Reference			Reference		
40–59	199 (47.38%)	1.496	0.422–5.302	0.533	1.234	0.346–4.401	0.746
≥60	144 (34.29%)	8.040	2.477–26.099	0.001	6.870	2.106–22.410	0.001
**Sex**							
Male	295 (70.24%)	Reference			Reference		
Female	125 (29.77%)	0.358	0.162–0.795	0.012	0.380	0.170–0.846	0.018
**Race**							
White	361 (85.95%)	Reference					
Black	24 (5.71%)	1.620	0.643–4.082	0.306			
Others/unknown	35 (8.33%)	0.190	0.026–1.378	0.100			
**Year of diagnosis**							
4–9	135 (32.14%)	Reference					
10–16	285 (67.86%)	0.843	0.458–1.554	0.585			
**Insurance**							
Uninsured/unknown/blank	80 (19.05%)	Reference					
Insured/no specifics	297 (70.71%)	1.109	0.563–2.183	0.765			
Any Medicaid	43 (10.24%)	1.630	0.605–4.391	0.334			
**Marital status**							
Married (including common law)	243 (57.86%)	Reference					
Other	177 (42.14%)	1.095	0.631–1.899	0.747			
**Primary site**							
Ventricle, NOS	203 (48.33%)	Reference					
Brain stem	128 (30.48%)	0.819	0.422–1.588	0.555			
Other	89 (21.19%)	1.139	0.577–2.251	0.707			
**Tumor size (cm)**							
<2	173 (41.19%)	Reference					
2–4	110 (26.19%)	0.922	0.46–1.843	0.818			
≥4	33 (7.86%)	1.427	0.538–3.786	0.475			
Unknown/blank	104 (24.76%)	1.324	0.662–2.649	0.428			
**Pathology**							
Benign	5 (1.19%)	Reference					
Subependymoma	411 (97.86%)	0.623	0.086–4.517	0.639			
Malignant	4 (0.95%)	0.932	0.058–14.916	0.960			
**Grade**							
Well differentiated	7 (1.67%)	Reference					
Moderately differentiated	5 (1.19%)	NA	NA	NA			
Undifferentiated	1 (0.24%)	NA	NA	NA			
Unknown	407 (96.90%)	0.392	0.095–1.617	0.195			
**Laterality**							
Left-origin of primary	51 (12.14%)	Reference					
Right-origin of primary	57 (13.57%)	0.850	0.274–2.638	0.779			
Not a paired site	296 (70.48%)	1.003	0.424–2.372	0.994			
Paired or bilateral	16 (3.81%)	0.578	0.070–4.806	0.612			
**Radiation**							
None/Unknown	406 (96.67%)	Reference					
Yes	14 (3.33%)	1.273	0.309–5.237	0.738			
**Vital status**							
Alive	368						
Dead	52						
**OS (M)**	48.50 (21–85)						

HR, hazard ratio; CI, confidence interval; OS, overall survival; NA, not available.

The median OS time was 51.5 months (IQR 17–89.75).

After PSM of 470 patients in unoperated versus GTR, 164 non-operative patients were matched with 164 GTR patients (**Tables 4**, **S2**). In the matched cohort, there was no significant difference in OS between the non-surgical and GTR groups (HR = 0.562; 95% CI, 0.299–1.054; *p* = 0.072; **Table 4**). In the multivariable regression analysis, sex (HR = 0.211; 95% CI, 0.065–0.684; *p* = 0.010; **Table 4**) was an independent risk prognostic factor for OS.

**Table 4:** The characteristics of 328 patients from the 470 patients grouped according to no surgery and GTR after PSM.

Characteristics	Value *N* = 328	Univariate analysis			Multivariate analysis		
		HR	95% CI	*p*-value	HR	95% CI	*p*-value
Primary site surgery							
No surgery	164 (50.00%)	Reference			Reference		
GTR	164 (50.00%)	0.549	0.292–1.030	0.062	0.562	0.299–1.054	0.072
**Age (years)**							
0–39	61 (18.60%)	Reference					
40–59	163 (49.70%)	NA	NA	NA			
≥60	104 (31.71%)	NA	NA	NA			
**Sex**							
Male	242 (73.78%)	Reference			Reference		
Female	86 (26.22%)	0.208	0.064–0.673	0.009	0.211	0.065–0.684	0.010
**Race**							
White	292 (89.02%)	Reference					
Black	16 (4.88%)	1.966	0.699–5.526	0.200			
Others/unknown	20 (6.10%)	NA	NA	NA			
**Year of diagnosis**							
4–9	110 (33.54%)	Reference					
10–16	218 (66.46%)	0.865	0.438–1.711	0.678			
**Insurance**							
Uninsured/unknown/blank	72 (21.95%)	Reference					
Insured/no specifics	228 (69.51%)	1.463	0.672–3.186	0.338			
Any Medicaid	28 (8.54%)	2.182	0.660–7.213	0.201			
**Marital status**							
Married (including common law)	186 (56.71%)	Reference					
Other	142 (43.29%)	1.124	0.606–2.083	0.711			
**Primary site**							
Ventricle, NOS	183 (55.79%)	Reference					
Brain stem	95 (28.98%)	1.237	0.608–2.516	0.557			
Other	50 (15.24%)	1.658	0.732–3.754	0.225			
**Tumor size (cm)**							
<2	121 (36.89%)	Reference					
2–4	81 (24.70%)	0.883	0.400–1.948	0.758			
≥4	25 (7.62%)	1.725	0.674–4.416	0.256			
Unknown/blank	101 (30.79%)	0.751	0.330–1.711	0.495			
**Pathology**							
Benign	5 (1.52%)	Reference					
Subependymoma	320 (97.56%)	0.626	0.086–4.567	0.644			
Malignant	3 (0.91%)	1.619	0.101–26.020	0.734			
**Grade**							
Well differentiated	6 (1.83%)	Reference					
Moderately differentiated	1 (0.30%)	NA	NA	NA			
Undifferentiated	0 (0.00%)	NA	NA	NA			
Unknown	321 (97.87%)	0.470	0.113–1.963	0.301			
**Laterality**							
Left-origin of primary	23 (7.01%)	Reference					
Right-origin of primary	41 (12.50%)	0.857	0.204–3.592	0.832			
Not a paired site	254 (77.44%)	0.900	0.275–2.941	0.861			
Paired or bilateral	10 (3.05%)	NA	NA	NA			
**Radiation**							
None/unknown	317 (96.65%)	Reference					
Yes	11 (3.35%)	1.525	0.367–6.328	0.561			
**Vital Status**							
Alive	287 (87.50%)						
Dead	41 (12.50%)						
**OS (M)**	49 (17–89.75)						

HR, hazard ratio; CI, confidence interval; GTR, gross total resection; OS, overall survival; NA, not available.

The median follow-up time was 49 months (IQR 20–91).

After PSM of 339 patients in unoperated versus STR, 92 non-operative patients were matched with 92 STR patients (**Tables 5**, **S3**). In the matched cohort, there was no significant difference in OS between the non-surgical and STR groups (HR = 0.765; 95% CI, 0.330–1.772; *p* = 0.532; **Table 5**). In the multivariable regression analysis, laterality (HR = 0.300; 95% CI, 0.106–0.847; *p* = 0.023; **Table 5**) was an independent risk prognostic factor for OS.

**Table 5:** The characteristics of 184 patients from the 339 patients grouped according to no surgery and STR after PSM.

Characteristics	Value *N* = 184	Univariate analysis			Multivariate analysis		
		HR	95% CI	*p*-value	HR	95% CI	*p*-value
Primary site surgery							
No surgery	92 (50.00%)	Reference					
STR	92 (50.00%)	0.765	0.330–1.772	0.532			
**Age (years)**							
0–39	36 (19.57%)	Reference					
40–59	88 (47.83%)	NA	NA	NA			
≥60	60 (32.61%)	NA	NA	NA			
**Sex**							
Male	139 (75.54%)	Reference					
Female	45 (24.46%)	0.033	0.000–2.379	0.118			
**Race**							
White	154 (83.70%)	Reference					
Black	9 (4.89%)	0.740	0.099–5.520	0.769			
Others/unknown	21 (11.41%)	0.329	0.044–2.454	0.278			
**Year of diagnosis**							
4–9	63 (32.24%)	Reference					
10–16	121 (65.76%)	1.590	0.596–4.241	0.354			
**Insurance**							
Uninsured/unknown/blank	43 (23.37%)	Reference					
Insured/no specifics	113 (61.41%)	2.058	0.715–5.927	0.181			
Any Medicaid	28 (15.22%)	1.865	0.443–7.860	0.396			
**Marital status**							
Married (including common law)	103 (55.98%)	Reference					
Other	81 (44.02%)	0.652	0.266–1.602	0.352			
**Primary site**							
Ventricle, NOS	88 (47.83%)	Reference					
Brain stem	53 (28.80%)	1.168	0.423–3.225	0.764			
Other	43 (23.37%)	1.376	0.498–3.802	0.538			
**Tumor size (cm)**							
<2	47 (25.54%)	Reference					
2–4	46 (25.00%)	1.224	0.373–4.010	0.739			
≥4	21 (11.41%)	1.496	0.357–6.266	0.582			
Unknown/blank	70 (38.04%)	1.374	0.449–4.205	0.578			
**Pathology**							
Benign	2 (1.09%)	Reference					
Subependymoma	180 (97.83%)	NA	NA	NA			
Malignant	2 (1.09%)	NA	NA	NA			
**Grade**							
Well differentiated	2 (1.09%)	Reference					
Moderately differentiated	2 (1.09%)	NA	NA	NA			
Undifferentiated	0 (0.00%)						
Unknown	180 (97.83%)	0.222	0.030–1.666	0.143			
**Laterality**							
Left-origin of primary	20 (10.87%)	Reference			Reference		
Right-origin of primary	23 (12.50%)	0.372	0.089–1.564	0.177	0.372	0.089–1.564	0.177
Not a paired site	131 (71.20%)	0.300	0.106–0.847	0.023	0.300	0.106–0.847	0.023
Paired or bilateral	10 (5.43%)	0.371	0.043–3.180	0.366	0.371	0.043–3.180	0.366
**Radiation**							
None/unknown	178 (96.74%)	Reference					
Yes	6 (3.26%)	0.048	0.000–9,009.212	0.624			
**Vital status**							
Alive	162 (88.04%)						
Dead	22 (11.96%)						
**OS (M)**	52.5 (16–93.75)						

HR, hazard ratio; CI, confidence interval; STR, subtotal resection; OS, overall survival; NA, not available.

The median follow-up time was 52.5 months (IQR 16–93.75).

After PSM of 344 patients in unoperated versus surgery NOS or excisional biopsy, 87 non-operative patients were matched with 87 patients with surgery NOS or excisional biopsy (**Tables 6**, **S4**). In the matched cohort, there was no significant difference in OS between the non-surgical and surgery NOS or excisional biopsy groups (HR = 0.596; 95% CI, 0.258–1.377; *p* = 0.225; **Table 6**). In the multivariable regression analysis, age (HR = 10.758; 95% CI,2.377–48.693; *p* = 0.002; **Table 6**) was an independent risk prognostic factor for OS.

**Table 6:** The characteristics of 174 patients from the 344 patients grouped according to no surgery and surgery NOS or excisional biopsy after PSM.

Characteristics	Value *N* = 174	Univariate analysis			Multivariate analysis		
		HR	95% CI	*p*-value	HR	95% CI	*p*-value
Primary site surgery							
No surgery	87 (50.00%)	Reference					
Surgery NOS or excisional biopsy	87 (50.00%)	0.596	0.258–1.377	0.225			
**Age (years)**							
0–39	36 (20.69%)	Reference			Reference		
40–59	94 (54.02%)	0.677	0.124–3.697	0.652	0.938	0.166–5.310	0.942
≥60	44 (25.29%)	7.204	1.664–31.197	0.008	10.758	2.377–48.693	0.002
**Sex**							
Male	119 (68.39%)	Reference					
Female	55 (31.61%)	0.697	0.258–1.884	0.477			
**Race**							
White	154 (88.51%)	Reference					
Black	10 (5.75%)	2.602	0.769–8.802	0.124			
Others/unknown	10 (5.75%)	NA	NA	NA			
**Year of diagnosis**							
4–9	69 (39.66%)	Reference			Reference		
10–16	105 (60.34%)	2.364	0.890–6.280	0.084	2.654	0.862–8.170	0.089
**Insurance**							
Uninsured/unknown/blank	35 (20.11%)	Reference			Reference		
Insured/no specifics	119 (68.39%)	1.452	0.468–4.503	0.519	0.925	0.277–3.090	0.899
Any Medicaid	20 (11.49%)	4.454	1.152–17.224	0.030	3.358	0.828–13.619	0.090
**Marital status**							
Married (including common law)	98 (56.32%)	Reference					
Other	76 (43.68%)	1.579	0.695–3.588	0.275			
**Primary site**							
Ventricle, NOS	89 (51.15%)	Reference					
Brain stem	45 (25.86%)	1.461	0.556–3.842	0.442			
Other	40 (22.99%)	1.630	0.591–4.496	0.345			
**Tumor size (cm)**							
<2	48 (27.59%)	Reference					
2–4	51 (29.31%)	1.429	0.507–4.026	0.499			
≥4	16 (9.20%)	1.178	0.237–5.844	0.841			
Unknown/blank	59 (33.91%)	0.930	0.300–2.888	0.901			
**Pathology**							
Benign	3 (1.72%)	Reference					
Subependymoma	169 (97.13%)	0.471	0.063–3.531	0.464			
Malignant	2 (1.15%)	1.702	0.105–27.593	0.708			
**Grade**							
Well differentiated	5 (2.87%)	Reference			Reference		
Moderately differentiated	1 (0.75%)	NA	NA	0.983	NA	NA	NA
Undifferentiated	1 (0.75%)	NA	NA	0.986	NA	NA	NA
Unknown	167 (95.98%)	0.180	0.042–0.778	0.022	0.280	0.058–1.358	0.114
**Laterality**							
Left-origin of primary	23 (13.22%)	Reference					
Right-origin of primary	24 (13.79%)	0.361	0.070–1.864	0.224			
Not a paired site	121 (69.54%)	0.515	0.187–1.416	0.199			
Paired or bilateral	6 (3.45%)	NA	NA	NA			
**Radiation**							
None/unknown	166 (95.40%)	Reference					
Yes	8 (4.60%)	2.767	0.639–11.978	0.173			
**Vital status**							
Alive	151 (86.78%)						
Dead	23 (13.22%)						
**OS (M)**	56.50 (21.75–97.25)						

HR, hazard ratio; CI, confidence interval; OS, overall survival; NA, not available.

The median follow-up time was 56.50 months (IQR 21.75–97.25).

## Discussion

Scheinker reported a case of a newly recognized tumor derived from the fourth subependymal zone in a 56-year-old man and firstly named subependymoma (8). To date, subependymoma was sporadically reported on case reports (4, 9–11) and accounted for 0.07%-0.7% of all brain tumors (9, 12). Subependymomas were brain neoplasms that tended to be benign, to be less aggressive, to grow slowly, and to be histologically classified as World Health Organization (WHO) grade 1 (13). D’Amico et al. reported a case that was diagnosed with subependymoma by pathological biopsy; CT and MRI confirmed no significant tumor progression after a 36-year follow-up, highlighting the extremely indolent nature of subependymoma (14). The pathogenesis of subependymoma may be related to potential precursor cells (13, 15). Zhiyong et al. reported that 43 patients with subependymoma were found in 60,000 cases of surgically intracranial tumors and the incidence of intracranial subependymoma was about 0.07%. The lesions were mostly located in lateral ventricles accounting for 65% of cases, followed by the fourth ventricle and third ventricle accounting for 19% and 7% of cases, respectively. Tumors were less common in the brain parenchyma and stem (2, 13). The occurrence of symptoms, such as initial clinical manifestations of increased intracranial pressure, was related to the disturbance of cerebrospinal fluid circulation caused by the tumor. Uncommon clinical symptoms including epilepsy, memory loss, ataxia, tremor, blurred vision, and subarachnoid hemorrhage have been reported in some cases (2, 7).

However, there is no prediction model for the OS of subependymoma and no large sample study about the impact of different surgical methods on patient prognosis.

We conducted a study for subependymoma based on the SEER database. The National Cancer Institute’s SEER database collected cancer diagnosis, treatment, and survival data for approximately 30% of the U.S. population. SEER database is an important population-based resource that has become a unique research resource for oncology practice in the United States. The SEER database had the following advantages: representative and universal responses to disease in the United States population, long data collection time, large number of cases, and collection of specific cancer outcomes.

However, the SEER database had the following limitations: individual-level data on specific cancer risks and treatments are incomplete. The accuracy and completeness of raw data collected from the registry needed to be improved. SEER database could not evaluate the progression-free survival (PFS) of tumors.

In this study, we combined the treatment data, follow-up data, and clinical and pathological data from 535 patients in the training group to construct a nomogram for the prediction of the OS of each patient.

### Independent prognostic predictors and nomogram

Nguyen et al. drew a conclusion that age < 40 years, female sex, and location within ventricles or near brain stem were positive factors with OS by analyzing 466 cases of intracranial subependymomas from 2004 to 2013 in the SEER database (16). The authors suggest that surgery remains a mainstay treatment. Like prior studies, our study supported that age and sex were significant independent predictors of OS. After our statistical analysis, the prognostic model constructed by age and sex was in good agreement with the reality.

However, whether in the training or subgroup cohort, we revealed that surgery, tumor size, and location were not independent prognostic factors for OS. This seems to challenge the choice of surgical treatment.

D’Amico et al. found that the presence of early malignant lesions in subependymoma cannot be confirmed by early imaging examination and drew a conclusion that early resection was preferred by immunohistochemical analysis of 31 patients with pathologically proven subependymomas (9).

However, some scholars also proposed that conservative treatment was the main treatment for subependymoma. Kammer et al. reviewed 33 cases and showed that subependymomas were usually symptomless; 29 patients were discovered by chance. Subependymoma with no obvious growth tendency seldom led to decompensation of cerebrospinal fluid circulation by blocking the interventricular foramen or Magendie foramen.

In other words, hydrocephalus was relatively rare in subependymoma, which recommended expectant treatment or longer imaging follow-up than other lesions at the same location (10). With a retrospective analysis of 13 patients with intracranial WHO grade 1 subependymoma from 1990 to 2015, Varma revealed that occasional intraventricular subependymoma could be treated conservatively with MRI monitoring. Because there was no significant change in disease during a mean follow-up of 46 months, long-term follow-up was not necessary (14). The author further expounded that hydrocephalus was the main complication of surgical treatment of hydrocephalic subependymoma (2, 14). The appeal suggests that conservative treatment was also an appropriate approach. This may seem counterintuitive. Due to the low degree of malignancy, there were few reports of death caused by subependymoma in a short period. Patients are more likely to die from accidents or other factors.

We recognized that the established nomogram had some value in evaluating patient prognosis and were inclined to use models to predict the prognosis of the subependymoma.

Although nomograms had certain predictive accuracy in the training and validation groups in our study, the treatment strategy still needed to be further improved through subsequent studies, considering the inherent limitations of the SEER databases.

This may not mean that surgery was meaningless for subependymoma. We conducted a further subgroup analysis of the benefits of surgical treatment.

### Subgroups analyses of different surgical methods

No large sample data analysis has reported the prognostic impact of different surgical methods for subependymoma.

Reviewing 466 patients with intracranial subependymoma, Nguyen et al. concluded that surgery was a significantly positive prognostic factor. However, the author further elaborated that GTR was not a significant prognostic factor and locations within “ventricles, NOS” or near “brain stem” were low-risk predictors factors for OS (16). Although there was the most extensive data analysis of intracranial subependymoma before 2017, the conclusion seemed to be counterintuitive.

Considering the importance of data quality, we screened the data of higher quality from the large sample and tried to make it equally comparable, through the application of statistical methods, such as eliminating incomplete data, univariate and multivariate analyses, PSM, and subgroup analysis.

To study the influence of different surgical methods on OS, we completed subgroup analyses through Cox regression analysis and PSM. Exhibited in **Tables 3** and **S1**, patients matched after PSM had no significant difference in OS between the non-surgical and surgical groups. In the multivariable regression analysis, age and sex were significant prognostic variables for OS. The subgroup analysis of non-surgical versus surgical groups confirmed this finding in the training cohort.

Surgical treatment, race, year of diagnosis, insurance, tumor location, tumor size, pathology, tumor grade, and radiation had no statistically significant differences in OS for subependymoma in our research.

However, it was diacritical that age, sex, and laterality were the significant prognostic variables for OS in the different matched subgroups of unoperated versus GTR, STR, and biopsy. However, it did not mean that conservative treatment had a better prognosis.

The study of Nguyen et al. included fewer patients, lacking subgroup analyses, not clear prognostic factors of different treatment modalities, and the confounding factors were not matched. Improving deficiencies of previous studies, we should not be more inclined to choose conservative or surgical treatment.

Considering the rarity of the disease, our study is a retrospective analysis of the largest sample size of subependymoma to date, taking advantage of the SEER database’s wide population coverage.

We predicted the 3-, 5-, and 10-year survival rates for subependymoma using a nomogram model based on age and sex as prognostic factors. Although the prognostic model performed well in the experimental and validation groups, the two prognostic factors might not be sufficient to clinical use.

Nonetheless, the information that we present might be useful to suggest potential hypotheses to be tested in the clinical research setting. Doctors needed to evaluate the indications, contraindications, and risks of surgery comprehensively, and then made recommendations based on the wishes of patients’ families.

We suggested that the following measures needed to be adopted before the clinical implementation. Due to the extremely indolent nature of subependymoma, longer follow-up time was required to assess the outcome of the operative treatment. We need to expand the sample further and include more prognostic variables, such as immunohistochemical information. Prospective multicenter randomized controlled studies of subependymoma were needed to develop models with greater sensitivity and specificity.

### Limitations

In our study, there was a particular patient selection bias based on the SEER database. Therefore, data quality is also a limitation of this study. Considering the rigor of the data, our study excluded patients with a survival time of less than 1 month, which might have skewed the results by excluding acute deaths from severe hydrocephalus without surgery. In addition, the median follow-up was only 56 months and the sample size was small after PSM. Longer follow-up and further multicenter studies with more sample sizes are needed.

## Conclusion

Age and sex were the independent prognostic variables for OS in intracranial subependymoma. According to our research, we should not be more inclined to choose conservative or surgical treatment. Nonetheless, the information that we present might be useful to suggest potential hypotheses to be tested in the clinical research setting.

## Data availability statement

The original contributions presented in the study are included in the article/**Supplementary Material**. Further inquiries can be directed to the corresponding author.

## Ethics statement

Ethical review and approval was not required for the study on human participants in accordance with the local legislation and institutional requirements. Written informed consent from the participants’ legal guardian/next of kin was not required to participate in this study in accordance with the national legislation and the institutional requirements.

## Author contributions

HC and ZZ: conception of the project. XP and YW: data reduction and screening. QL and HJ: statistical analysis and processing of data. ZZ, HC, and YW: writing and revising article. All authors contributed to the article and approved the submitted version.

## Funding

This research project was funded by the Key Science Research Foundation of Traditional Chinese Medicine of Zhejiang Province of China (2019ZA089).

## Conflict of interest

The authors declare that the research was conducted in the absence of any commercial or financial relationships that could be construed as a potential conflict of interest.

## Publisher’s note

All claims expressed in this article are solely those of the authors and do not necessarily represent those of their affiliated organizations, or those of the publisher, the editors and the reviewers. Any product that may be evaluated in this article, or claim that may be made by its manufacturer, is not guaranteed or endorsed by the publisher.

## Supplementary material

Supplementary Table 1 The χ^2^, Fisher’s exact or Mann-Whitney U test was used to inspect the differences of the non-surgical and surgical groups before and after matching. Age, insurance and pathology were analyzed by using the Mann-Whitney U test. The grade was analyzed by using Fisher’s exact test.

Supplementary Table 2 The χ^2^, Fisher’s exact or Mann-Whitney U test was used to inspect the differences of the no surgery and GTR before and after matching. Age, insurance and pathology were analyzed by using the Mann-Whitney Utest. The grade was analyzed by using Fisher’s exact test.

Supplementary Table 3 The χ^2^, Fisher’s exact or Mann-Whitney U test was used to inspect the differences of the no surgery and STR in before and after matching. Age, insurance and pathology were analyzed by using the Mann-Whitney U test. The grade was analyzed by using Fisher’s exact test.

Supplementary Table 4 The χ^2^, Fisher’s exact or Mann-Whitney U test was used to inspect the differences of the no surgery and Surgery NOS or excisional biopsy before and after matching. Age, insurance and pathology were analyzed by using the Mann-Whitney U test. The grade was analyzed by using Fisher’s exact test.
