# Supplementary material for: Clinical independent prognostic factors and overall survival prognostic nomogram for intracranial subependymoma: A SEER population-based analysis 2004–2016
Source: Front Oncol. 2022 Aug 22;12:939816. doi: 10.3389/fonc.2022.939816 (PMC9442051; doi:10.3389/fonc.2022.939816)
Supplement: Supplementary Table 1 — The χ2, Fisher’s exact or Mann-Whitney U test was used to inspect the differences of the non-surgical and surgical groups before and after matching. Age, insurance and pathology were analyzed by using the Mann-Whitney U test. The grade was analyzed by using Fisher’s exact test. [file DataSheet_1.pdf]

SUPPLEMENTARY MATERIAL

TABLE S1 | The χ^2, Fisher's exact or Mann-Whitney U test was used to inspect the differences of the non-surgical and surgical groups before and after matching. Age, insurance and pathology were analyzed by using the Mann-Whitney U test. The grade was analyzed by using Fisher's exact test.

Characteristics	Before PSM				After PSM			
	No surgery	Surgery	χ^2	*P*-value	No surgery	Surgery	χ^2	*P*-value
**Age (Y)**				0.001				0.744
0-39	42	84			42	35		
40-59	100	231			91	108		
60~	101	109			77	67		
**Sex**			3.757	0.053			0.285	0.594
Male	161	311			145	150		
Female	82	113			65	60		
**Race**			1.973	0.373			0.784	0.676
White	207	377			183	178		
Black	16	21			12	12		
Others/unknown	20	26			15	20		
**Year of diagnosis (Y)**			9.072	0.003			1.845	0.174
04-09	66	164			61	74		
10-16	177	260			149	136		
**Insurance**				0.220				0.616
Uninsured/ unknown/blank	37	94			36	44		
Insured/No specifics	184	283			154	143		
Any Medicaid	22	47			20	23		
**Marital Status**			7.326	0.007			6.103	0.013
Married (including common law)	127	267			109	134		
Other	116	157			101	76		
**Primary Site**			0.947	0.623			5.970	0.051
Ventricle, NOS	131	216			114	89		
Brain stem	67	132			57	71		
Other	45	76			39	50		
**Tumor Size(cm)**			101.674	<0.001			32.085	<0.001
<2	134	82			104	69		
2~4	37	165			34	76		
4~	10	52			10	23		
Unknown/blank	62	125			62	42		
**Pathology**				0.690				0.739
Benign	3	3			3	2		
Subependymoma	238	418			205	206		
Malignant	2	3			2	2		
**Grade**				<0.001				0.099
Well differentiated	3	47			3	4		
Moderately differentiated	0	10			0	5		
Undifferentiated	0	1			0	1		
Unknown	240	366			207	200		
**Laterality**			7.223	0.065			0.301	0.960
Left-origin of primary	35	42			26	25		

Right-origin of primary	36	50			28	29		
Not a paired site	162	322			147	149		
Paired or Bilateral	10	10			9	7		
**Radiation**			1.341	0.247			0.000	1.000
None/Unknown	236	404			203	203		
Yes	7	20			7	7		
**Total**	243	424			210	210		

PSM, propensity score matching

TABLE S2 | The χ^2, Fisher's exact or Mann-Whitney U test was used to inspect the differences of the no surgery and GTR before and after matching. Age, insurance and pathology were analyzed by using the Mann-Whitney U test. The grade was analyzed by using Fisher's exact test.

Characteristics	Before PSM				After PSM			
	No surgery	GTR	χ^2	*P*-value	No surgery	GTR	χ^2	*P*-value
**Age (Y)**				0.001				0.316
0-39	42	41			33	28		
40-59	100	134			71	92		
60~	101	52			60	44		
**Sex**			4.201	0.040			0.063	0.802
Male	161	170			122	120		
Female	82	57			42	44		
**Race**			4.178	0.124			0.000	1.000
White	207	207			146	146		
Black	16	10			8	8		
Others/unknown	20	10			10	10		
**Year of diagnosis (Y)**			7.176	0.007			2.681	0.102
04-09	66	88			48	62		
10-16	177	139			116	102		
**Insurance**				0.015				0.026
Uninsured/ unknown/blank	37	59			30	42		
Insured/No specifics	184	149			115	113		
Any Medicaid	22	19			19	9		
**Marital Status**			4.647	0.031			4.024	0.045
Married (including common law)	127	141			84	102		
Other	116	86			80	62		
**Primary Site**			0.488	0.783			4.352	0.114
Ventricle, NOS	131	123			99	84		
Brain stem	67	67			39	56		
Other	45	37			26	24		
**Tumor Size(cm)**			67.099	<0.001			24.589	<0.001
<2	134	50			77	44		
2~4	37	91			25	56		
4~	10	25			8	17		
Unknown/blank	62	61			54	47		
**Pathology**				0.527				0.998
Benign	3	2			3	2		
Subependymoma	238	222			159	161		
Malignant	2	3			2	1		
**Grade**				<0.001				1.000
Well differentiated	3	29			3	3		
Moderately differentiated	0	4			0	1		
Undifferentiated	无	无			无	无		
Unknown	240	194			161	160		
**Laterality**			12.798	0.005			3.810	0.283
Left-origin of primary	35	16			12	11		
Right-origin of primary	36	24			20	21		
Not a paired site	162	183			124	130		

Paired or Bilateral	10	4			8	2		
**Radiation**			0.419	0.517			0.094	0.759
None/Unknown	236	218			159	158		
Yes	7	9			5	6		

PSM, propensity score matching; GTR, gross total resection.

TABLE S3 | The χ^2, Fisher's exact or Mann-Whitney U test was used to inspect the differences of the no surgery and STR in before and after matching. Age, insurance and pathology were analyzed by using the Mann-Whitney U test. The grade was analyzed by using Fisher's exact test.

**Characteristics**	**Before PSM**				**After PSM**			
	**No surgery**	**STR**	χ^2	***P*-value**	**No surgery**	**STR**	χ^2	***P*-value**
**Age (Y)**				0.106				0.478
0-39	42	24			13	23		
40-59	100	39			50	38		
60~	101	33			29	31		
**Sex**			2.449	0.118			0.029	0.864
Male	161	72			69	70		
Female	82	24			23	22		
**Race**			1.022	0.600			0.159	0.924
White	207	80			77	77		
Black	16	5			5	4		
Others/unknown	20	11			10	11		
**Year of diagnosis (Y)**			4.214	0.040			0.603	0.437
04-09	66	37			29	34		
10-16	177	59			63	58		
**Insurance**				0.583				0.194
Uninsured/ unknown/blank	37	21			24	19		
Insured/No specifics	184	56			57	56		
Any Medicaid	22	19			11	17		
**Marital Status**			2.349	0.125			2.669	0.102
Married (including common law)	127	59			46	57		
Other	116	37			46	35		
**Primary Site**			2.008	0.366			4.737	0.094
Ventricle, NOS	131	44			47	41		
Brain stem	67	33			20	33		
Other	45	19			25	18		
**Tumor Size(cm)**			47.294	<0.001			13.142	0.004
<2	134	17			30	17		
2~4	37	31			16	30		
4~	10	16			6	15		
Unknown/blank	62	32			40	30		
**Pathology**				0.777				1.000
Benign	3	0			2	0		
Subependymoma	238	96			88	92		
Malignant	2	0			2	0		
**Grade**				0.005				0.747
Well differentiated	3	4			1	1		
Moderately differentiated	0	3			0	2		
Undifferentiated	240	89			无	无		
Unknown					91	89		
**Laterality**			1.478	0.687			0.512	0.916
Left-origin of primary	35	10			10	10		
Right-origin of primary	36	12			12	11		
Not a paired site	162	70			64	67		

Paired or Bilateral	10	4			6	4		
**Radiation**			0.362	0.547			0.000	1.000
None/Unknown	236	92			89	89		
Yes	7	4			3	3		

PSM, propensity score matching; STR, subtotal resection.

TABLE S4 | The χ^2, Fisher's exact or Mann-Whitney U test was used to inspect the differences of the no surgery and Surgery NOS or excisional biopsy before and after matching. Age, insurance and pathology were analyzed by using the Mann-Whitney U test. The grade was analyzed by using Fisher's exact test.

Characteristics	Before PSM				After PSM			
	No surgery	Biopsy	χ^2	*P*-value	No surgery	Biopsy	χ^2	*P*-value
**Age (Y)**				0.013				0.636
0-39	42	19			19	17		
40-59	100	58			43	51		
60~	101	24			25	19		
**Sex**			0.137	0.711			0.665	0.415
Male	161	69			62	57		
Female	82	32			25	30		
**Race**			1.230	0.541			0.904	0.636
White	207	90			75	79		
Black	16	6			6	4		
Others/unknown	20	5			6	4		
**Year of diagnosis (Y)**			4.413	0.036			0.024	0.877
04-09	66	39			34	35		
10-16	177	62			53	52		
**Insurance**				0.826				0.296
Uninsured/ unknown/blank	37	14			23	12		
Insured/No specifics	184	78			52	67		
Any Medicaid	22	9			12	8		
**Marital Status**			5.746	0.017			5.981	0.014
Married (including common law)	127	67			41	57		
Other	116	34			46	30		
**Primary Site**			0.878	0.645			7.781	0.020
Ventricle, NOS	131	49			47	42		
Brain stem	67	32			15	30		
Other	45	20			25	15		
**Tumor Size(cm)**			56.046	<0.001			18.203	<0.001
<2	134	15			33	15		
2~4	37	43			14	37		
4~	10	11			7	9		
Unknown/blank	62	32			33	26		
**Pathology**				0.713				0.658
Benign	3	1			2	1		
Subependymoma	238	100			83	86		
Malignant	2	0			2	0		
**Grade**				<0.001				1.000
Well differentiated	3	14			3	2		
Moderately differentiated	0	3			0	1		
Undifferentiated	0	1			0	1		
Unknown	240	83			84	83		
**Laterality**			1.105	0.776			0.951	0.813
Left-origin of primary	35	16			11	12		
Right-origin of primary	36	14			13	11		
Not a paired site	162	69			59	62		

Paired or Bilateral	10	2			4	2		
**Radiation**			2.998	0.083			0.524	0.469
None/Unknown	236	94			84	82		
Yes	7	7			3	5		

PSM, propensity score matching.
